# Improving clinical trial design for Duchenne muscular dystrophy

**DOI:** 10.1186/s12883-015-0408-z

**Published:** 2015-08-26

**Authors:** Luciano Merlini, Patrizia Sabatelli

**Affiliations:** Laboratory of Musculoskeletal Cell Biology, Istituto Ortopedico Rizzoli, IRCCS, Via Di Barbiano 1/10, 40136 Bologna, Italy; CNR National Research Council of Italy, Institute of Molecular Genetics, Bologna, Italy

**Keywords:** Duchenne muscular dystrophy, Dystrophin evaluation, Clinical trial, Corticosteroid treatment, Exon skipping, Splice modulation

## Abstract

**Background:**

Currently, the most promising therapies for Duchenne muscular dystrophy (DMD) are exon skipping and stop codon read-through, two strategies aimed at restoring the expression of dystrophin. A phase 3 clinical trial with drisapersen, a drug designed to induce exon 51-skipping, has failed to show significant improvement of the primary outcome measure, the six-minute walk test.

**Discussion:**

Here, we review some key points that should be considered when designing clinical trials for these new therapies. First, younger patients have more functional abilities and more muscle fibers to preserve than older patients and therefore are better subjects for trials designed to demonstrate the success of new treatments. Second, the inclusion of patients on corticosteroids both in the treatment and placebo groups is of concern because the positive effect of corticosteroids might mask the effect of the treatment being tested. Additionally, the reasonable expectation from these therapies is the slowing of disease progression rather than improvement. Therefore, the appropriate clinical endpoints are the prolongation of the ability to stand from the floor, climb stairs, and walk, not an increase in muscle strength or function. Hence, the time frames for the detection of new dystrophin, which occurs within months, and the ability to demonstrate a slowing of disease progression, which requires years, are strikingly different. Finally, placebo-controlled trials are difficult to manage if years of blindness are required to demonstrate a slowing of disease progression. Thus, accelerated/conditional approval for new therapies should be based on surrogate biochemical outcomes: the demonstration of de novo dystrophin production and of its beneficial effect on the functional recovery of muscle fiber.

**Summary:**

These data suggest that clinical trials for DMD patients must be adapted to the particular characteristics of the disease in order to demonstrate the expected positive effect of new treatments.

## Background

Currently, the most promising therapies for Duchenne muscular dystrophy (DMD) include two small-molecule approaches, exon skipping and stop codon read-through, both of which aim to restore the expression of dystrophin from the mutant endogenous gene. With respect to exon skipping, the preclinical studies in *mdx* mouse models of DMD have demonstrated very impressive dose-dependent production of dystrophin and a therapeutic effect on dystrophic muscles [1]. In DMD patients, two chemistries targeting dystrophin exon 51, drisapersen, a 2’-O-methyl-phosphorothioate (2OMePS), and eteplirsen, based on phosphoramidate morpholino (PMO), both elicited the expected exon 51 skipping and local dystrophin restoration following intramuscular injection [2, 3]. However, the 186-patient phase 3 clinical trial with drisapersen (NCT01254019) failed to show a significant improvement of the primary outcome measure, the six-minute walk test (6MWT) [1]. In a subsequent phase 2 study, the boys in the continuous drisapersen group demonstrated transient improvements in 6MWT compared with those receiving the placebo but not in the other clinical outcome measures, including the time to stand, time to run 10 m, and time to climb stairs [4]. As in previous studies [5], there was very limited evidence of drug-induced dystrophin production in patient’s muscles [6].

Eteplirsen has been associated with more evident *de novo* dystrophin production than drisapersen in two studies [6–8]. In the open-label extension study, there was also stabilization of the clinical outcome in a subset of patients [7]. However, it remains to be seen whether eteplirsen can maintain a significant long-term clinical benefit [1]. In the second study [8], two patients treated with eteplirsen lost ambulation despite a consistent increase in dystrophin-positive fibers.

In a phase 2b trial that included 174 patients, ataluren, designed to permit stop codon read-through, was associated with a marginally significant improvement in the 6MWT compared with the placebo [9]. However, this drug has shown no clear evidence of facilitating dystrophin restoration [10], and the scoring method used in the trial was considered very subjective [11–14].

The failure of the only phase 3 study of antisense oligonucleotides (drisapersen) performed so far has raised considerable discussion about the validity of dystrophin as a biomarker and the 6MWT as an outcome measure [1, 6, 11, 12, 15]. However, other critical points have not been considered. In this article, we review some of the characteristics of DMD that should influence the trial design for DMD treatments. We first review recent literature documenting the possibility of earlier clinical diagnosis of DMD and the indications that the disease has already a progressive course during infancy. Next we document the beneficial effects of corticosteroid treatment to highlight the fact that the inclusion of patients on corticosteroids in both the treatment and control groups may be problematic. We then focus on the importance of choosing clinical outcome measures that match the type of improvement expected from treatment. The clinical endpoints that are appropriate if the effect of treatment is amelioration are not appropriate if the expected impact is slowing disease progression. Finally, we propose a way to comply with the requirement that a correlation be shown between dystrophin expression and clinical outcomes, realizing that the production of dystrophin can take months but slowing of disease progression requires years to demonstrate.

## Discussion

### DMD: early onset and progressive course

The X-linked, progressive, muscle-wasting disease DMD is caused by mutations in the gene encoding dystrophin. DMD is a fatal neuromuscular disease, affecting 1 in 3500–6000 live male births [16, 17]. In affected boys, a diagnosis can be suspected at birth on the basis of markedly elevated levels of serum creatine kinase (CK), and eventually confirmed with genetic or dystrophin analysis. In the past, most patients were diagnosed around the age of 5 and observed to subsequently show some improvement in motor skills, albeit at a slower rate than normal boys (the “honeymoon” period in DMD) [18]. Recently, the clinical course of the disease has been better defined using assessment scales validated in infants from birth. Significant deficits in gross and fine motor function are already present in infants and young boys [19]. Motor function further declines within the first 3 years of life compared with age-matched peers [20]. Boys with DMD have a progressive muscle weakness, with a 50-60 % drop in strength by age 5 [20]. The loss of clinical milestones occurs in a predictable descending order: loss of standing from the floor, loss of stairs climbing, and loss of ability to walk independently [21]. Respiratory, orthopedic, and cardiac complications emerge in wheelchair-bound DMD patients [22]. Progressive scoliosis develops in over 90 % of patients as a combined result of wheel-chair dependence, paralysis of the extensor muscles [23], contractures, and growth spurts. Lung function increases up to the age of 10–12, plateaus, and then decreases, with an estimated loss of 10 % per year of forced vital capacity (FVC) [24]. Without treatment, death occurs in the early- to mid-teens due to cardiorespiratory compromise [17]. The provision of non-invasive mechanical ventilation, assisted coughing, and cardio-protective medication allows survival into the late twenties and thirties [25].

The early onset and progressive clinical course of the disease is matched by the laboratory findings. The CK level, a biochemical marker of muscle necrosis, is 50- to 100-fold above normal in affected fetuses, at birth, and during the first year, decreases around the time when affected boys become wheel chair-bound (age 8–10), and only approaches normal values in the very late stages of the disease [26, 27]. Abnormal findings on muscle biopsy have been detected from DMD fetuses as early as the second trimester of pregnancy [26] and in DMD infants at 40 days [28] and 4 months of age [29].

### Clinical trials: starting time

The design of clinical trials in DMD should take in consideration that the disease has an early onset and a progressive, predictable course [21]. The diagnosis of DMD is now feasible much earlier than in the past. The marked elevation of CK is already present at birth. A florid dystrophic process is evident in the muscle biopsy of affected newborns. Within their first 3 years of life, DMD infants and young boys show measurable deficits in gross and fine motor function. In addition, their motor function declines within the first 3 years of life compared with age-matched peers. Having recognized that the dystrophic process is biologically and clinically present and measurable at an early age, there is no reason to delay treatment until the age of 7, when muscle weakness and myofiber loss are already advanced. Older boys with DMD have less muscle to rescue by exon skipping or any other approach and more fibrous and fatty connective tissue between myofibers, reducing contraction efficiency and possibly impairing regeneration [1].

### Beneficial effect of corticosteroids

The first scientific evidence of the beneficial effect of corticosteroids in DMD was documented over 40 years ago by Drachman [30]. Since then, several other studies have demonstrated the efficacy of corticosteroid therapy in delaying the loss of independence and autonomous ambulation and in maintaining adequate pulmonary function [31]. Currently, corticosteroids are the gold standard treatment for muscle weakness in ambulant children with DMD [31]. The most common daily dosage regimes are 0.75 mg/kg/day prednisone/prednisolone and 0.9 mg/kg/day deflazacort [31]. Other studies using corticosteroids with various combinations of daily, alternate-day, or cyclical prednisone treatment [32–35] have also demonstrated benefit in functional parameters. Corticosteroids are now routinely prescribed in most countries for DMD patients; however, there is no consensus on the optimal age to initiate treatment and the optimal dose and dosage schedule [31, 36].

Despite the fact that most of the different dosage schedules claim to be effective in improving muscle strength and function, none has been shown to maintain this result with time [31, 33, 37–43]. All long-term studies, independent of dose and dosage schedule, have shown that after a variable period of “improvement,” patients invariably lose muscle strength and function, although at a slower rate than patients not treated with corticosteroids.

In summary it is now widely recognized that long-term corticosteroid therapy (1) prolongs ambulation, (2) reduces the need for spinal surgery, (3) reduces cardiopulmonary dysfunction, (4) delays the need for mechanical ventilation, and (5) increases survival and quality of life of DMD patients [20, 31, 35, 41, 42, 44]. Recent findings also indicate that early use of corticosteroids is associated with significant advantages over delaying its use [20, 28, 35].

The inclusion of patients on corticosteroids both in the treatment and placebo groups is of concern because the positive effect of corticosteroids may mask the effect of the treatment being tested. On the other side, currently, corticosteroid therapy is the only recognized effective treatment for DMD patients; thus, avoiding its use may be difficult or possibly ethically unacceptable. However, it seems that parents of DMD patients are willing to accept more uncertainty and take greater risk early on due to the predictable outcome of the disease [45]. In addition, according to a WMA press release in 2001 [46], a placebo-controlled trial may be ethically acceptable even if proven therapy is available if there are compelling and scientifically sound methodological reasons justifying the need for such a study to determine the efficacy of a therapeutic method. The clinical trials of the new emerging therapies have followed two different and questionable approaches regarding the inclusion of patients on corticosteroids. In the exon-skipping trials, the patients were randomized to receive the test agent or placebo in addition to their current corticosteroid therapy [4, 8]. In this add-on trial design, efficacy is established only for the combination treatment rather than the added new drug as monotherapy. In an add-on trial, it is desirable to recruit patients on the same maintenance therapy or at least to stratify based on the different treatments. In one study [8], 12 patients were receiving 2 different corticosteroids at 4 different doses and dosage regimens. In the ataluren trial, 70 % of patients in the two arms of the study were receiving corticosteroids [8] with very different doses and regimens, thereby raising questions as to the validity of the interpretation of clinical outcomes.

### Clinical trials: treatment expectations

**Fig. 1:**
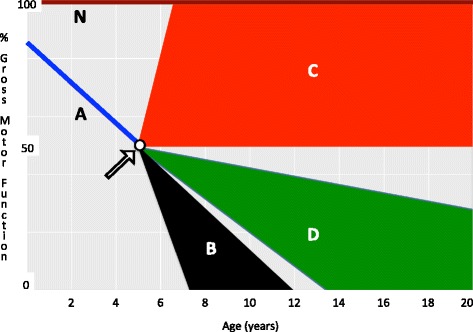
DMD course and treatment outcomes. A DMD boy beginning treatment at age 5 (arrow) when he has already lost some motor function/muscle strength (line A) compared with normal peers (line N). Without treatment, he will continue to deteriorate, finally loosing ambulation between 7–12 years of age (black triangle – B). The red area (C) represents the outcome of a treatment able to arrest (base of the red trapezoid) or partially or completely to restore (leg of the trapezoid) lost function. The green area (D) represents the outcome of a treatment able to slow the progression of the disease with loss of autonomous ambulation after the age of 14 years

A clinical trial design should reflect the type of clinical benefit that is expected from treatment and the time required to demonstrate benefit. The positive effects of intervention on the course of a chronic progressive disease like DMD may involve 1) arresting the course of disease with or without restoration of the lost function or 2) slowing the progression of the disease (Fig. 1).

The choice of clinical trial endpoints should take into consideration these two different expectations. If the expectation of the intervention is arresting the course of the disease, that is, stopping the loss of muscle fibers, then an increase or conservation of muscle strength or muscle function are suitable endpoints to evaluate efficacy. However, these endpoints are not appropriate for an intervention that is only expected to slow disease progression. In this case, muscle strength (maximal isometric muscle force, FVC) or muscle function (6MWT) may show a transient improvement at some point in time; however, they are expected to deteriorate with time, although at a slower rate compared with no treatment. Some endpoints appropriate for demonstrating a slowed disease progression are 1) survival to death, 2) survival to death or any respiratory intervention or 3) prolongation of independent walking. It is evident that all of these endpoints require a long period of treatment/observation, particularly if treatment is started early during the course of the disease. This point was well demonstrated by a prospective, long-term, open-label study of treatment with alternate-day corticosteroids in five 2- to 4-year-old DMD patients. This study had prolongation of the ability to walk as primary outcome measure [28, 35, 47]. At the last follow-up, four of these five patients, aged 16 to 18, were fully ambulant, and three of them could still climb stairs. Muscle strength, measured with a myometer, increased slightly from age 5 to 8 and then declined steadily. This progressive decline was particularly evident and dramatic for the knee extensors, with an average of 122 N at age 8, 80 N at age 12, 55 N at age 16, and 44 N at age 18 [35]. This study suggests that long-term corticosteroid treatment is effective in prolonging function but not in recovering muscle strength and lost function and its early use seems appropriate. In summary, at best, the effect of corticosteroid treatment is to slow the progressive course of the disease [33, 35, 48, 49]. A reasonable and primary expectation for trials of new DMD therapies is the preservation of independent ambulation beyond the age of 12 [50]. During the typical time frame of a clinical trial, one year, increasing the distance walked during a 6MWT by 30 or more meters or reducing the time required for a 10-m walk by a few seconds does not guarantee that patients will be able to walk longer, the definition of treatment success. The easiest way to evaluate the preservation of ambulation is to use the timed 10-m test to determine the velocity expressed in m/s. In this way, all the patients can be scored, including those who are no longer able to walk.

The next question is “What type of clinical result is foreseeable for any treatment aimed at de novo production of dystrophin in DMD patients?” A complete, long-lasting restoration of dystrophin around all of the residual myofibers in DMD patients is too optimistic. Becker muscular dystrophy (BMD) patients with in-frame deletions including exon 51 typically have a milder phenotype and longer life span than DMD patients [51, 52]; however, in these patients, the shortened dystrophin is present from birth. A treatment begun in a DMD boy after age 7 will at best result in a phenotype somewhere between that of a moderately severe BMD patient and a less progressive DMD patient. BMD is clinically defined as a patient remaining ambulant at least age 16 or later [17]. Hence, any DMD treatment should be considered effective if it is able to prolong ambulation at least beyond the age of 16, a result that has already been achieved in some DMD patients with early or long-term corticosteroid therapy [35, 38, 43].

### Dystrophin evaluation

Currently, the quantification of newly produced dystrophin is the earliest endpoint to evaluate and to determine the efficacy of antisense oligonucleotides or stop codon read-through treatments [6]. Promising tools such as melanocytes, spectroscopy, and other miRNAs have yet to be validated [1, 15, 53, 54]. The methods that are presently used to evaluate the presence of dystrophin, western blot (WB) and immunofluorescence (IF), are actually semi-quantitative methods that determine the signal of dystrophin normalized to an internal marker and expressed relative to control. This implies that only the samples examined in the same experiment can be compared (same session IF or loaded together in the same gels used for a WB) and that it is incorrect to compare results obtained with samples tested at different times or in different laboratories.

There is no doubt that standard operating procedures for the detection of dystrophin [1, 55–57] are needed; however, two considerations are relevant on this regard. First, the procedures used to date are all acceptable as semi-quantitative methods, and yet, all of the methods are questionable as quantitative methods. Second, if we grant that the fragment of muscle analyzed reflects the situation in the whole muscle, still it remains to be established the relationship between the amount of newly synthesized dystrophin and the functionality of the cell.

### Dystrophin expression and clinical outcome

The approval of exon skipping and splice modulation treatments as therapies for DMD requires that a correlation be shown between dystrophin expression and clinical outcomes. Although dystrophin can be produced within months, prolongation of walking, the desired clinical outcome, can take years to be demonstrated, especially if treatment is started early. Because of this challenge, accelerated approval should be based on a surrogate biochemical outcome (e.g., demonstration of de novo dystrophin production in muscle). Most important, if we grant that the fragment of muscle analyzed reflects the situation in the whole muscle, once it has been shown that newly produced dystrophin is detectable in response to a treatment designed to restore dystrophin, its effect on the functional recovery of muscle fiber should be demonstrated. A second biological end point is thus needed. For example, in a baseline muscle biopsy, a lack of dystrophin and a cell dysfunction (e.g., secondary deficiency of the dystrophin-associated glycoprotein complex, mitochondrial dysfunction, defective autophagy, etc.) are shown. In the post-treatment muscle biopsy, a significant increase of dystrophin [6] and correction of the cell dysfunction should be demonstrated. If a treatment satisfies both the biological endpoints, application for accelerated approval could be made. Placebo-controlled trials are also not an option if a decade or more of blindness is needed to show a slowing of disease progression in the treated group compared with the placebo group.

## Summary

We suggest ways to overcome the problems associated with the previous and on-going DMD clinical trial designs. First, younger patients have more muscle fibers to rescue and functional abilities to preserve and are thus more suitable patients in which to demonstrate long-lasting success of the new treatments. Second, the inclusion of patients on corticosteroids in both the treatment and placebo groups should be avoided because the positive effect of corticosteroids might mask the effect of the treatment being tested. Third, the reasonable expectation from these therapies is the slowing of disease progression, not long-lasting improvement. Therefore, the appropriate clinical endpoints are prolongation of the ability to stand from the floor, climb stairs, and walk rather than an increase of muscle strength or function. Fourth, the production of dystrophin can occur within months, but the slowing of disease progression requires years to demonstrate. Because of this gap in time, the accelerated/conditional approval of new DMD therapies should be based on surrogate biochemical outcomes: demonstration of de novo dystrophin production and its beneficial effect on the functional recovery of muscle fiber.

## Abbreviations

6MWT: 6-minute walk test
BMD: Becker muscular dystrophy
CK: Creatine kinase
DMD: Duchenne muscular dystrophy
FVC: Forced vital capacity
IF: Immunofluorescence
WB: Western blot
